# Simultaneous Induction of Non-Canonical Autophagy and Apoptosis in Cancer Cells by ROS-Dependent ERK and JNK Activation

**DOI:** 10.1371/journal.pone.0009996

**Published:** 2010-04-02

**Authors:** Chew Hooi Wong, Kartini Bte Iskandar, Sanjiv Kumar Yadav, Jayshree L. Hirpara, Thomas Loh, Shazib Pervaiz

**Affiliations:** 1 Department of Physiology, Yong Loo Lin School of Medicine, National University of Singapore, Singapore, Singapore; 2 NUS Graduate School for Integrative Sciences and Engineering, National University of Singapore, Singapore, Singapore; 3 Department of Otorhinolaryngology, National University Hospital System, Singapore, Singapore; 4 Cancer and Stem Cell Biology Program, Duke-NUS Graduate Medical School, Singapore, Singapore; 5 Singapore-MIT Alliance, Singapore, Singapore; INMI, Italy

## Abstract

**Background:**

Chemotherapy-induced reduction in tumor load is a function of apoptotic cell death, orchestrated by intracellular caspases. However, the effectiveness of these therapies is compromised by mutations affecting specific genes, controlling and/or regulating apoptotic signaling. Therefore, it is desirable to identify novel pathways of cell death, which could function in tandem with or in the absence of efficient apoptotic machinery. In this regard, recent evidence supports the existence of a novel cell death pathway termed autophagy, which is activated upon growth factor deprivation or exposure to genotoxic compounds. The functional relevance of this pathway in terms of its ability to serve as a stress response or a truly death effector mechanism is still in question; however, reports indicate that autophagy is a specialized form of cell death under certain conditions.

**Methodology/Principal Findings:**

We report here the simultaneous induction of non-canonical autophagy and apoptosis in human cancer cells upon exposure to a small molecule compound that triggers intracellular hydrogen peroxide (H_2_O_2_) production. Whereas, silencing of beclin1 neither inhibited the hallmarks of autophagy nor the induction of cell death, Atg 7 or Ulk1 knockdown significantly abrogated drug-induced H_2_O_2_-mediated autophagy. Furthermore, we provide evidence that activated extracellular regulated kinase (ERK) and c-Jun N-terminal kinase (JNK) are upstream effectors controlling both autophagy and apoptosis in response to elevated intracellular H_2_O_2_. Interestingly, inhibition of JNK activity reversed the increase in Atg7 expression in this system, thus indicating that JNK may regulate autophagy by activating Atg7. Of note, the small molecule compound triggered autophagy and apoptosis in primary cells derived from patients with lymphoma, but not in non-transformed cells.

**Conclusions/Significance:**

Considering that loss of tumor suppressor beclin 1 is associated with neoplasia, the ability of this small molecule compound to engage both autophagic and apoptotic machineries via ROS production and subsequent activation of ERK and JNK could have potential translational implications.

## Introduction

It is now well established that chemotherapy-induced reduction in tumor load is a function of apoptotic cell death, orchestrated by intracellular caspase proteases. However, the effectiveness of some of these therapies is blunted by mutations affecting specific effectors genes controlling and/or regulating apoptotic signaling, such as epigenetic silencing of caspase 8, downregulation of pro-apoptotic proteins Bax and Apaf-1, as well as upregulation of the anti-apoptotic proteins of the Bcl-2 and IAP families. Therefore, there has been a surge of activity around identification of novel pathways of cell death, which could function in tandem with or in the absence of efficient apoptotic machinery. In this regard, recent evidence has highlighted the existence of a novel, caspase-independent pathway, termed autophagy, which is activated in response to growth factor deprivation or upon exposure to genotoxic compounds[1]. Whereas, the jury is still out on the functional relevance of this pathway in terms of its ability to serve as a stress response or a truly death effector mechanism, recent evidence seems to support that autophagy is a specialized form of cell death under certain conditions[2], [3], [4].

Among the several effector mechanisms involved in the control and regulation of cell death pathways, including apoptosis and autophagy, is the cellular redox status. The redox status of the cell is determined by the balance between the rates of production and breakdown of reactive oxygen and/or nitrogen species (ROS; RNS)[5], such as superoxide anion (O_2_^−^), hydrogen peroxide (H_2_O_2_), hydroxyl radical (OH), nitric oxide (NO) and hypochlorus acid (HOCl)[6]. We have previously shown that tumor cell response to death stimuli is a function of cellular redox status, and stimuli, in particular death-inducing compounds, that induce a significant increase in intracellular H_2_O_2_ facilitate death execution[7], [8], [9], [10] [11], [12], [13], [14], [15].

Interestingly, ROS have also been shown as strong signals for the activation of mitogen activated protein kinase (MAPK) family of signaling proteins comprising of C-jun N-terminal Kinase (JNK), p38 and ERK[16]. The MAPK family members are activated in a 3-tier kinase cascade comprising of MAPK kinase kinase (MAPKKK), MAPK kinase (MAPKK) and MAPK[17]. Sustained activation of JNK has been directly linked to an increase in intracellular ROS production[18], and a possible mechanism could be through inactivation of MAPK Phosphatases (MKP)[6]. Of note, an increase in intracellular ROS as well as activation of MAPK have been demonstrated during autophagic execution[19], [20].

Autophagy has been well recognized as the garbage disposal of the cell, being mainly involved in the sequestration of plasma membrane and long-lived organelles into autophagosomes, which eventually fuse with the lysosomes for degradation and recycling of nutrients[21], [22]. More importantly, persistent autophagy in response to cellular stress states serves as a potent death signal[23], [24], [25], as in the case of therapy-induced autophagy, a specific non-apoptotic death pathway triggered upon exposure to chemotherapeutic compounds[26]. The latter forms the basis for the identification of Type II cell death, characterized by excessive autophagosome formation[27], [28]. Paradoxically, basal level of autophagy induced by stresses such as hypoxia and growth factor withdrawal tips the cellular fate towards survival [29], [30], [31].

The activation of the canonical autophagy pathway is critically under the control of the BH-3 only Bcl-2 interacting protein, Beclin1[32]. Notably, recent evidence has unraveled a novel autophagic cell death pathway wherein Beclin1 is completely dispensable[33]. This could be of paramount importance as the execution of non-canonical autophagy in cancer cells bearing a Beclin1 knockout phenotype, could represent a novel and effective strategy to induce cancer cell death[34].

Here we report the mechanism of cell death induced in human tumor cells by a novel small molecule compound, 1,3-dibutyl-2-thiooxo-imidazolidine-4,5-dione(C1). Exposure of human colorectal carcinoma and a variety of other tumor cell lines to C1 resulted in cell death with morphological and biochemical features consistent with non-canonical autophagy and apoptosis. Furthermore, we highlight the seminal involvement of early ROS production and ERK and JNK activation upstream of the autophagic and apoptotic pathways. This is a novel report highlighting the simultaneous induction of Beclin1-independent autophagy and apoptosis in tumor cells by a small molecule compound, which could have tremendous clinical implications, considering the relatively high incidence of Beclin1 loss in human cancers.

## Materials and Methods

### Reagents and Chemicals

The pan-caspase inhibitor, ZVAD-FMK, and the caspase 3 and 9 inhibitors (DEVD-fmk and VEHD-fmk) were obtained from Alexis Biochemicals (Lausen, Switzerland). The JNK inhibitor (SP600125), the ERK inhibitor (PD98059), bovine catalase, Earle's Balanced Salt Solution (EBSS) medium, rapamycin, diethyldithiocarbamate (DDC), E64D, pepstatin-A, 3-Methyladenine, C2-Ceramide, N-acetyl-cysteine, crystal violet, and MTT were purchased from Sigma Aldrich (St. Louis, MO). Superoxide dismutase (SOD) was purchased from Calbichem (Merck KGaA, Darmstadt, Germany). Tumor necrosis factor-alpha (TNF-α) was obtained from Upstate (Millipore, MA).

### Tumor Cell Lines

HCT116 colorectal carcinoma cells were generously provided by Dr. Bert Vogelstein (The Johns Hopkins University School of Medicine, Baltimore, MD) and maintained in McCoy 5A (Gibco Invitrogen Corporation, Carlsbad, CA) supplemented with 10% fetal bovine serum FBS), 1% L-glutamine, and 1% S-Penicillin (Hyclone, Thermo Scientific, Waltham, MA) in a 37°C incubator with 5% CO_2_. HeLa cervical carcinoma, A549 small cell lung carcinoma, M14 melanoma, SHEP1 and SHSY5Y neuroblastoma cell lines were obtained from ATCC and maintained in DMEM (Hyclone) supplemented with 10% FBS. MCF-7, T47D, and MB-MDA231 breast cancer cell lines were from ATCC and cultured in RPMI (Hyclone) supplemented with 10% FBS. HK-6, C666-1 nasopharyngeal carcinoma cell lines, gifted by Dr. Lo Kwok-wai (The Chinese University of Hong Kong) and Dr. Hsieh Wen-son (Johns Hopkins Singapore), were maintained in RPMI supplemented with 10% FBS.

### Transfection with pGFP-rLC3 and siRNA

For transient expression, cells were transfected with 8 µg of pGFP-LC3 or pCINeoEV or pCINeo+Cat plasmids in Optimem1™ medium (Invitrogen Corporation, Carlsbad, CA) using the Superfect transfection reagent (Qiagen, Valencia, CA) according to the manufacturer's instructions. For knockdown of gene expression, 50 nM siRNA (beclin- 1 siRNA, or JNK1/2 siRNA, or ERK1/2 siRNA, or Atg7 siRNA, or ULK-1 siRNA) was transfected into cells in Optimem1™ medium using the Dharmafect1 reagent (Dharmacon) according to the manufacturer's instructions.

### Cell viability and tumor colony forming assays

Cell viability following drug exposure was determined by the MTT assay as described previously(14). For colony forming assays, 10,000 cells were plated in petri dishes and grown for 2 weeks. The plates were then stained with crystal violet solution (Sigma Aldrich, St. Louis, MO) and colonies were scored manually as described previously [35].

### Propidium Iodide staining for DNA fragmentation

Cells were treated with C1 for the indicated time points as described in figure legends prior to propidium iodide staining for DNA content analysis as described previously (14). Histogram data indicating percentage of cells with sub-diploid DNA are shown and are mean ± SD of three independent observations.

### Flow cytometric analysis of intracellular ROS

Cells were loaded with 5 µmol/L of the redox sensitive dye 5-(and-6)-chloromethyl-2-,7-dichlorofluorescin diacetate (Molecular Probes, Invitrogen Corporation) as described previously(14) and analyzed by flow cytometry (Coulter EPICS Elite ESP). Detection of intra-mitochondrial O_2_^−^ was performed by loading cells with MitoSox™ RED MITOCHODNRIAL O_2_^−^ INDICATOR (Molecular Probes, Invitrogen Corporation) as described previously and analyzed by flow cytometry (Coulter EPICS Elite ESP). At least 10,000 events were analyzed.

### Isolation of mitochondria

HCT116 cells treated with C1 for 6, 12 and 24 hr were subjected to mitochondrial extraction as described elsewhere(14). The mitochondrial pellets were lysed in standard 1xRIPA lysis buffer and the supernatants were used as the cytosolic fractions.

### Immunofluorescence analysis of pGFP-rLC3 and acridine orange

Analysis of LC3 was performed using pGFP-rLC3 transfected cells by immunofluorescence microscopy. Following transfection with pGFP empty vector or pGFP-rLC3, cells were incubated with C1 for 6 to 24 hours and visualized by a fluorescent microscope (Eclipse TE2000-S, Nikon) using excitation wavelength of 488 nm and emission wavelength of 525 nm. For the visualization of lysosomes, cells were treated for 6 hours and then stained with 2.5 µg/ml acridine orange (Molecular Probes, Invitrogen Corporation) for 10 mins at 37°C, and analyzed for both, green and red flurorescence, using fluorescence microscopy as mentioned above.

### Electron microscopy

Cells were fixed overnight in 2.5% glutaraldelhyde in 0.1 M phosphate buffer (pH 7.2), before being post-fixed in 1% OsO4 for 1 hour. Next, cells were dehydrated in ethanol series and embedded in Spurr's resin. Ultra thin sections were stained with uranyl acetate and lead citrate and observed under a JEOL JEM-1230 transmission electron microscope.

### Cytotoxicity assay for primary cells from lymphoma tissues

Primary tumor tissue from patients with T or B cell lymphoma were obtained from consenting patients at the National University Hospital in accordance with the approved IRB protocol. Tissues were minced and strained through MACS separation filter (Miltenyi Biotec) to obtain single cell suspensions. Lymphocytes were obtained following Ficoll Hypaque gradient centrifugation. Cell viability of primary lymphoma/200 µl/well in 96 well plates was determined by MTT assay after exposure to 25 and 50 µg/ml of C1 for 24 hrs. MTT assay was assessed as described in the previous section.

### Western blot analysis

Whole cell protein extracts were isolated using 1X RIPA lysis buffer (50 mM Tris-HCl, pH 7.4, 150 mM NaCl, 0.25% deoxycholic acid, 1% NP-40, 1 mM EDTA and protease inhibitors (Calbiochem, San Diego, CA). Equal amounts of protein from the total cell lysates (30 to 120 µg/lane) were separated by sodium dodecyl sulfate (10%, 12% or 15%) polyacrylamide gel electrophoresis gels (SDS-PAGE; BioRad Laboratories), transferred to PVDF membrane (BioRad Laboratories) using wet transfer (BioRad Laboratories) and blotted with primary antibodies specific for Bax, p62, LC3, Caspase 9, β-actin, GAPDH, ULK1 (Santa Cruz Biotechnology), Beclin1, ATG7, ATG12, ATG5, cytochrome C, JNK, p-JNK, c-JUN, p-c-JUN, Bid (Cell Signaling Technology), Caspase 3 (Upstate, Millipore Corporation), Caspase 8, Smac, anti-poly(ADP-ribose) polymerase (BD Pharmingen) and Catalase (Calbiochem) and probed with respective secondary isotype specific antibodies tagged with horseradish peroxidase (Thermo Scientific Pierce, Rockford, IL). Bound immuno-complexes were detected using WEST PICO Chemiluminescence substrate (Thermo Scientific Pierce, Rockford, IL).

### Synthesis and analysis of the small molecule compound C1

The small molecule compound 1,3-dibutyl-2-thiooxo-imidazolidine-4,5-dione, herein referred to as C1, was synthesized as follows: Oxalyl chloride was added to 1,3-dibutyl-2-thiourea (10 mM) in anhydrous ether in a round bottom flask under stirring. The reaction mixture was stirred for 1 to 2 hours at ambient temperature and then poured into saturated NaHCO_3_. The product was extracted with 3X ethyl acetate. The ethyl acetate layer was then washed with distilled water and then brine water. Ethyl acetate was then dried with anhydrous Mg_2_SO_4_ and removed under reduced pressure. The purification through flash chromatography (ethyl acetate:hexane) afforded the yellow oil product. The oily product was solidified in a refrigerator. The compound was then analyzed by ^1^HNMR, ^13^C NMR and MS and results are presented as follows: *Name: 1,3-dibutyl-2-thiooxo-imidazolidine-4,5-dione; Color: Orange; FT-IR (in CH_2_Cl_2_): 2875-2960 cm^-^ (Aliphatic CH), 1770 (C = O), 1410 (C = S); ^1^HNMR (in CDCL_3_): δ = 0.95 9 (t, J = 7.3 Hz, 6H, 4′-CH_3_), 1.34 (sext, J = 7.7 Hz, 4H, 3′-CH_2_), 1.67 (quint, J = 7.2 Hz, 4H, 2-CH_2_) 3.93 (t, J = 7.5 Hz, 4H, 1′-CH_2_); ^13^C NMR (in CHCl_3_): δ = 13.54 (C4′), 19.90 (C-3′), 29.72 (C-2′), 41.83 (C-1′), 155.35 (C-4.5), 180.63 (C-2); Mass m/z (%): 242 (100) [M^+^], 209 (26) [M+ -HS], 187(22); MF C_11_H_18_N_2_O_2_S calculated 242.34, Found 243.34.*

*Yield: 95%*

### Statistical Analysis

All experiments were performed at least 3 times unless otherwise stated. Experimental differences were tested for statistical significance using Student's *t*-test. *P* value of <0.05 was considered as significant.

## Results

### C1 induces cell death and MOMP in human tumor cells

**Figure 1:**
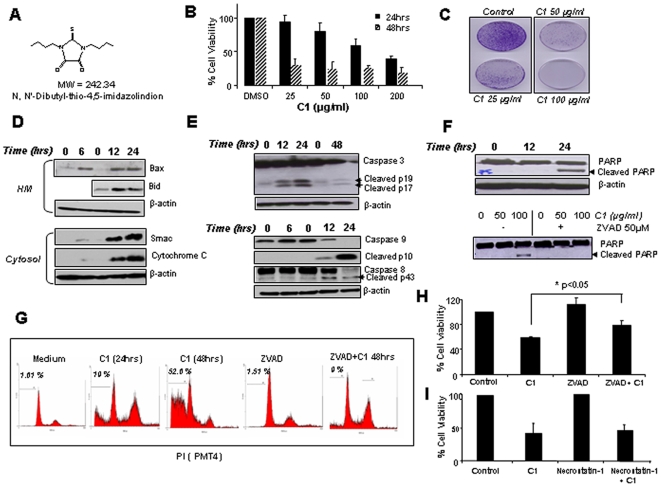
Exposure of HCT116 cells to C1 induces apoptotic hallmarks and MOMP. (**A**) Chemical structure and molecular weight of the compound C1. (**B**) Cells were treated with increasing concentrations of C1 (25 µg/ml to 100 µg/ml) for 18 hours and cell survival was assessed by the MTT assay. (**C**) Following exposure to C1 as in (**B**), 10,000 cells were seeded onto 100 mm petri dishes for assessment of colony formation. (**D**) Cells were treated with C1 (100 µg/ml) for 6, 12 and 24 hours, and sub-cellular fractions were subjected to western blot analysis. (**E**) Lysates from cells treated with C1 (100 µg/ml) for was probed for processing of caspases 3, 9 and 8. (**F**, **G**, **H**) Cells were treated with C1 (100 µg/ml for 18 h) in the presence or absence of zVAD-fmk (50 µM) for 12 and 24 hours and (**F**) lysates were probed for PARP cleavage and (**G**) cell cycle profiles were obtained by PI staining and (**H**) survival was assessed by the MTT assay. (**I**) Cells were pre-incubated with Necrostatin-1 (50 µM for 1 hour) before exposure to C1 (100 µg/ml for 18 h) and survival was assessed by the MTT assay.

Exposure of tumor cells to increasing concentrations (25–200 µg/ml) of C1 resulted in a dose-dependent decrease in cell viability at 24 hours with the LD50 of ∼100 µg/ml (Figure 1B). Furthermore, colony formation assay showed a significant reduction in clonogenic ability at 25 and 50 µg/ml and a complete cessation of colony formation at 100 µg/ml (Figure 1C). Results also showed that incubation of cells with C1 triggered mitochondrial outer membrane permeabilization (MOMP) as evidenced by the translocation of cytochrome c and Smac, and the reciprocal translocation of Bax and Bid to the mitochondria (Figure 1D). In addition, we obtained evidence for efficient processing of caspases 3, 8 and 9, and cleavage of the caspase 3 substrate PARP in total cell lysates from cells following C1 treatment, which was rescued in the presence of zVAD-fmk (Figure 1E, F). Analysis of cell cycle revealed 20% of the cells with sub-diploid DNA content (sub-G1 population) following 24 hours treatment, which was significantly increased (52%) upon incubation for 48 hours and blocked in the presence of the pan-caspase inhibitor (Figure 1G). Intriguingly, zVAD only partially provided protection form C1-induced cell death (Figure 1H). These data indicated that C1-induced cell death might involve caspase-independent pathways. To investigate that, we first evaluated the effect of the necrosis inhibitor, necrostatin, on C1-induced cell death. Results showed that while necrostatin could effectively abrogate Tumor Necrosis Factor-α-induced cell death (Supplementary Figure S1A), it had no effect on C1-induced cell death, thereby ruling out the involvement of necrosis in this model (Figure 1I).

### Induction of Beclin1-independent autophagy by C1

**Figure 2:**
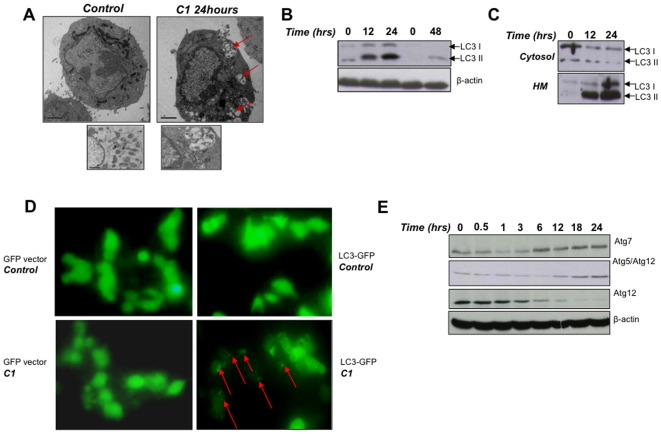
C1 induces atypical autophagic phenotype with upregulation of critical *Atg* genes. (**A**) HCT116 cells were treated with C1 (100 µg/ml) for 24 hours, fixed and viewed under an electron microscope (Magnification × 40,000). Arrows indicate the presence of autophagosomes. (**B**) Lysates of cells treated with C1 (100 µg/ml) were probed for LC3II. (**C**) Western blotting analysis of LC3II within cytosolic and mitochondrial fractions of cells following exposure to C1. (**D**) Cells were transiently transfected with GFP-Vector or LC3-GFP for 48 hours, exposed to C1 for 24 hours and then analyzed using a fluorescence microscope. Arrows point to the punctate staining indicating LC3 II aggregation into autophagosomes. (Mag: 40,000×). (**E**) Following C1 exposure, cell lysates were probed for Atg7, Atg5 and Atg12.

Next, electron microscopic analysis of cell morphology was performed following exposure to C1. Intriguingly, exposure of cells to C1 (100 µg/ml for 24 hours) resulted in the formation of autophagosomes and autophagic vacuoles (Figure 2A). In addition, the increased expression of MAP1 LC3II (herein referred to as LC3II) was observed in a time-dependent manner (Figure 2B). Of particular note, we identified for the first time LC3II enrichment in the heavy membrane fractions following drug treatment (Figure 2C). This may indicate the presence of mitochondrial engulfment by the autophagic vacuoles. Moreover, cells transfected with LC3-GFP displayed a diffuse pattern, while exposure to C1 resulted in a green punctate staining, indicative of LC3II accumulation within the autophagosomal membranes (Figure 2D). In addition, a significant increase in expression of Atg7 and Atg5 together with a reciprocal decrease in Atg12 expression was observed upon exposure (6–24 hours) of cells to C1, indicating Atg5-Atg12 conjugation (Figure 2E).

Next we examined if there was sufficient autophagic flux upon exposure to C1. Autophagic flux is crucial in determining whether the autophagic cargo and assembly finally reaches the lysosomes and is subsequently degraded. One of the ways to determine autophagic flux is pre-treatment with lysosomal inhibitors, E64D and pepstatin A, prior to the addition of the stimulus. Lysosomal inhibitors increase LC3 II formation, partly by blocking the autophagosomal-lysosomal fusion. Therefore, we investigated the effect of lysosomal inhibition on C1-induced LC3II formation. Our results showed that the inhibitors increased LC3II turnover in the cells at early time points (6 hours), however, there was no further increase upon longer incubation (24 hours) with the compound (Supplementary Figure S1E). We then checked for the expression level of p62/SQSTM1, a marker of autophagic flux[36]. The levels of p62 decreased upon C1 treatment at early time points indicative of efficient autophagy, however the levels increased subsequently (12–24 hrs; Supplementary Figure S1F), which could imply the possibility of aberrant autophagic flux. The latter observation was further supported by evidence of lysosomal rupture, assayed by acridine orange, which fluoresces red in acidic compartments such as the lysosomes and green in neutral pH. An increase in green flurorescence with a reciprocal decrease in red flurorescence was observed in cells upon exposure to C1, indicating rupture of the lysosomes (Supplementary Figure S1F).

**Figure 3:**
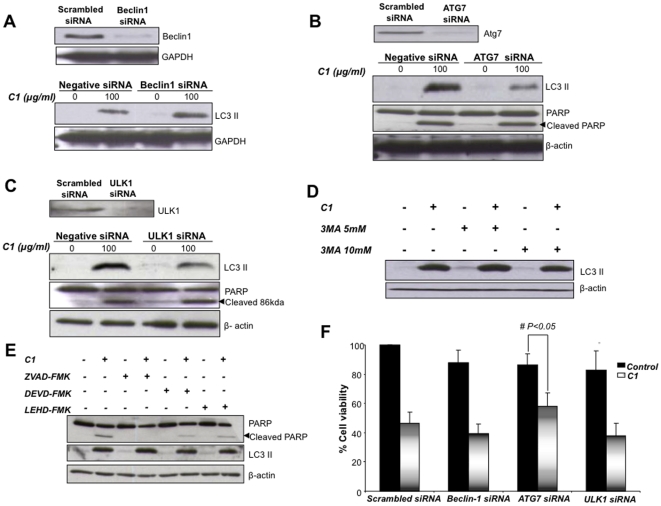
C1 induced autophagy is independent of Beclin1. (**A**), (**B**) and (**C**) Cells were transiently transfected with siRNA against Beclin1, or ATG7 or ULK1 for 48 hours followed by exposure to C1 (100 µg/ml) for 24 hours. Whole cell lysates were then probed for LC3II. (**D**) Cells were pre-incubated with 3-MA (5 or 10 mM) for 1 hour before exposure to 100 µg/ml of C1 for 18 hours. Lysates were then immuno-blotted with anti-LC3. (**E**) Cells were pre-treated with ZVAD-fmk (50 µM), caspase 3 inhibitor (50 µM) or caspase 9 inhibitor (50 µM) prior to the addition of 100 µg/ml of C1 for 18 hours. Lysates were then immuno-blotted with anti-PARP and anti-LC3 antibody. (**F**) Cells were transfected with siRNA against Beclin-1 or Atg7 or ULK1 and then exposed to 100 µg/ml of C1 for 18 hours. Cell viability was assessed by the MTT assay as described in Materials and Methods.

In order to assess the role of Beclin1 in C1-induced autophagy, RNAi-mediated silencing of *Beclin1* was performed. Knock down of *Beclin1* neither inhibited LC3II accumulation induced by C1 nor could rescue cells from the death triggering activity of the small molecule compound (Figure 3A). Notably, *Beclin1* silencing could effectively abrogate serum starvation-induced LC3II formation, demonstrating the classical role of Beclin1 in starvation-induced autophagy (Supplementary Figure S1C). In contrast to *Beclin1* silencing, si*Atg7* resulted in a significant decrease in LC3II accumulation induced upon C1 exposure while the apoptotic signal (PARP cleavage) remained unchanged (Figure 3B). Taken together, these data provide strong evidence that exposure of HCT116 cells to C1 triggers non-canonical autophagy, but involves the intermediacy of the ubiquitin E1-like enzyme Atg7. Corroborating these findings are results obtained with gene knockdown of the UNC-51 like kinase (ULK1; mammalian homolog of yeast Atg1), which similarly inhibited LC3II formation in this model (Figure 3C). Furthermore, *a priori* treatment of cells with 3-MA (5 or 10 mM) had virtually no effect on LC3 II accumulation induced by C1 (Figure 3D). Importantly, pre-treatment of HCT116 cells with ZVAD-fmk, caspase 3 inhibitor or caspase 9 inhibitor did not alter the accumulation of LC3II I induced by C1, indicating that signals governing apoptosis do not impact the autophagic signaling pathway (Figure 3E). More importantly, silencing of Atg7 significantly protected HCT116 cells from C1-induced reduction in cell viability, indicating that autophagic signal could be an effective cell death trigger (Figure 3F). As Beclin1 was not involved in C1-induced autophagy, knockdown of Beclin1 naturally did not alter the cell death response (Figure 3F). However, ULK silencing also had no significant effect on cell death (Figure 3F), thus arguing in favor of compensatory effects exerted by other prominent *Atg* genes.

**Figure 4:**
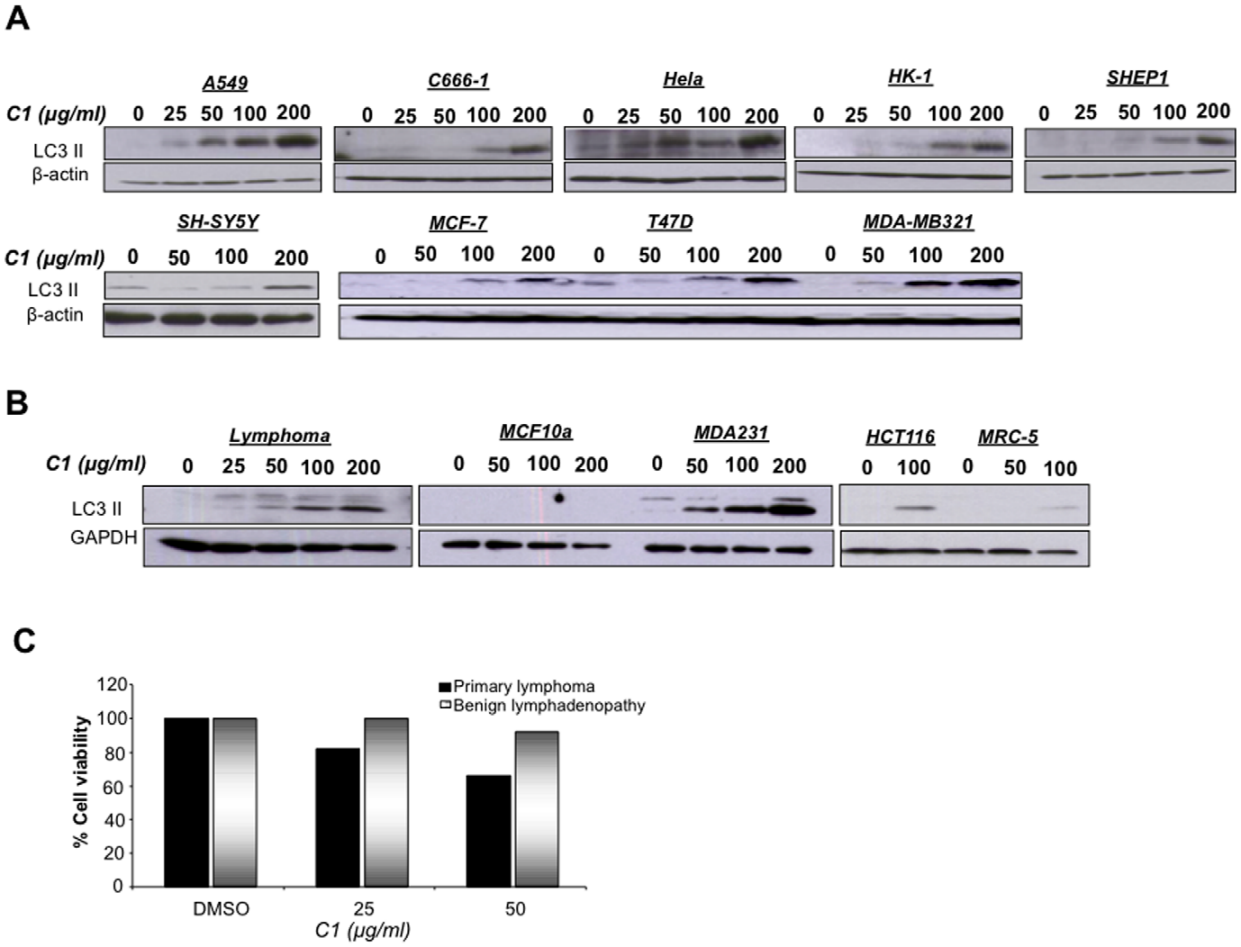
C1 induces apoptosis and autophagy in primary culture of lymphoma tissues, but not in benign tissue and non-carcinogenic cell lines. (**A**) Primary cultures of lymphoma and benign tissues were grown in 6-well plates and exposed to C1 at 25 and 50 µg/ml for 24 hours and cell survival was assessed by the MTT assay. (**B**) Primary lymphoma cells, MDA-MB-231 breast cancer cells, MCF10a mammary epithelial cells, HCT116 colorectal cells and MRC-5 human lung fibroblasts were being treated with C1 at various doses for 18 hours. Cell lysates were then being immuno-blotted with anti-LC3 with GAPDH as loading control. (**C**) Tumor cells from different lineages were treated with various concentrations of C1 for 24 hours and total cell lysates were probed for LC3 II and β-actin.

To provide evidence that the autophagy-inducing effect of this small molecule compound was not restricted to colorectal carcinoma cell line, LC3II formation was assessed in 9 other human cancer cell lines of varied origins. Indeed, an increase in LC3II formation was observed in all cell lines tested, albeit at different drug concentrations (Figure 4A). It should be noted that, similar to HCT116 cells, these cell lines were also sensitive to apoptosis induction by C1 (data not shown). To further explore the translational relevance of these data obtained with established cell lines, we next tested the effect of C1 on non-transformed cells (MCF-10A and MRC lines) as well as on primary cells obtained from patients with lymphomas. Unlike cancer cells, non-transformed cells did not show any sign of autophagy in response to C1, compared to HCT116 cells and MDA-MB-231 cells (Figure 4B). Furthermore, a representative sample from a patient with clinical lymphoma clearly elicited strong LC3II formation in response to drug exposure (Figure 4B). More importantly, exposure of primary cells derived from lymphoma patients (n = 12) showed dose-dependent sensitivity to C1, whereas cells from non-cancerous lymph nodes were relatively refractory to the treatment (Figure 4C). These data clearly established that the small molecule compound C1 triggered and cell death in a variety of cancer cell lines and in primary cells, while sparing non-transformed cells.

### Intracellular ROS controls C1-induced autophagy and apoptosis

**Figure 5:**
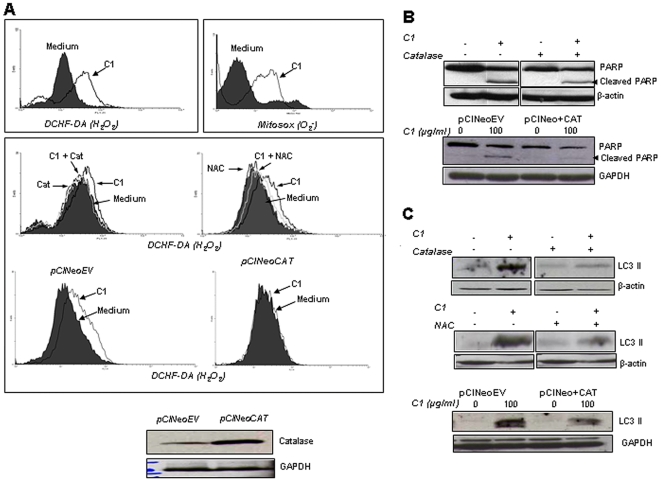
C1 induces early mitochondrial ROS production. In all, 1×10^6^ cells were incubated with 100 µg/ml of C1 for 3 hours and (**Ai**) intra-mitochondrial O_2_^-^ was determined using the fluorescent dye MitoSox™ RED Mitochondrial O_2_^-^ Indicator and intracellular H_2_O_2_ was detected by DCHF-DA loading and analyzed by flow cytometry. (**Ai**) Cells were pre-incubated with catalase (7000 units/ml) or NAC (200 µM) for 1 hour before treatment with C1 (100 µg/ml for 3 hours) and intracellular H_2_O_2_ was determined. (**Aii**) Cells were transiently transfected with 8 µg of pCINeoEV or pCINeo+CAT for 48 hours (**Aiii**) and treated with C1 (100 µg/ml for 3 hours) and intracellular H_2_O_2_ was determined (**Aii**). Cells were pre-incubated with catalase (7000 units/ml for 1 hour) or were transiently transfected with pCINeoEV or pCINeo+CAT before exposure to C1 (100 µg/ml for 24 hours), and total cell lysates were immunoblotted for (**B**) PARP cleavage and (**C**) LC3II accumulation.

Having clearly established the ability of C1 to simultaneously induce autophagy and apoptosis in cancer cells, we set out to investigate the molecular mechanism(s) underlying this biological activity. First, we assessed the effect on intracellular ROS production using two different fluorescent probes (MITOSOX™ RED and CM-DCHF-DA). Indeed, exposure of cells to C1 resulted in a significant increase in intracellular ROS production as measured by the H_2_O_2_-sensitve probe CM-DCHF-DA as well as an increase in intra-mitochondrial O_2_^−^ production (Figure 5A). Pre-incubation of cells with the ROS scavenger N-acetyl cysteine (NAC;200 µM) or the H_2_O_2_ scavenger, catalase (7000 units/ml), completely blocked the increase in CM-DCHF-DA fluorescence, strongly suggesting that the ROS species involved is H_2_O_2_ (Figure 5B). Corroborating these findings, overexpression of human catalase was found to abrogate C1-induced ROS production, assessed by CM-DCHF-DA staining (Figure 5A). Interestingly, catalase pre-incubation as well as transient overexpression of plasmid containing human catalase gene also inhibited C1-induced PARP cleavage, a marker of caspase 3 activation (Figure 5B). A similar inhibitory effect of catalase (exogenous addition as well as overexpression) and NAC was observed on LC3II accumulation induced by C1 (Figure 5C). These data strongly implicate intracellular H_2_O_2_ as the upstream stimulus that controlled both autophagy and apoptosis in this model.

To investigate the role of superoxide in our model, we pre-incubated the cells with the O_2_^−^ scavenger, superoxide dismutase (SOD), before the addition of C1. Interestingly, a significant increased in PARP cleavage was observed in the presence of SOD, thereby implicating H_2_O_2_ in the apoptotic signaling triggered by C1 (Supplementary Figure S1D). Conversely, increasing O_2_^−^ production by pre-incubation with the SOD inhibitor, diethyldithiocarbamate (DDC), increased LC3II formation (Supplementary Figure S1D). These findings suggest that tilting the intracellular balance between these two ROS species could profoundly affect cellular response to apoptosis or autophagy.

### ROS-induced JNK and ERK activation dictates the simultaneous induction of autophagy and apoptosis

**Figure 6:**
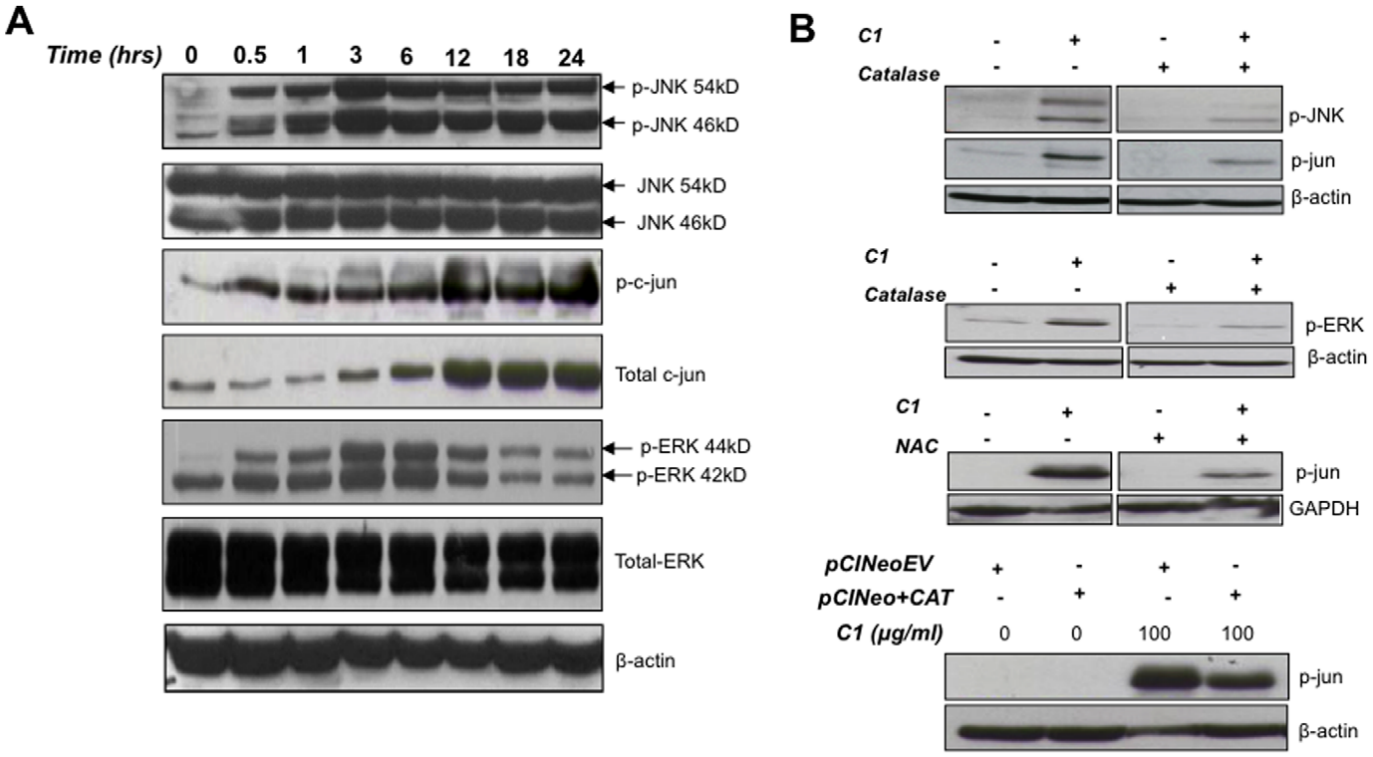
ROS-mediated JNK, c-JUN and ERK activation upon exposure to C1. (**A**) Cells were exposed to 100 µg/ml of C1 for the indicated time points and (**B**) cells were pre-incubated for 1 hour with 7000 U/ml of catalase or 200 µM NAC or transiently transfected with pCINeoEV or pCINeo+CAT before exposure to C1 (100 µg/ml for 3 hours). Total cell lysates were immuno-blotted for detection of phospho-JNK, total JNK, phospho-c-JUN, phospho-ERK and total ERK.

ROS has been shown as a potent regulator of MAP kinase family members [6], [37], [38]. To gain further insight into the mechanism of ROS-induced autophagy and apoptosis, we next assessed the involvement of the two critical MAPK family members, JNK and ERK. We provide evidence for robust activation of JNK (phosphorylation) as early as 30 minutes following exposure to the ROS inducing compound, which was sustained for 24 hours after the stimulus (Figure 6A). In addition, phosphorylation of ERK was detected in a time-dependent manner, which peaked at 3 hours and subsided at late time points (Figure 6A). Total c-Jun levels as well as phosphorylation at Ser-73 residue were detected as early as 30 minutes upon C1 treatment and persisted throughout the time course of 24 hours (Figure 6A).

Intrigued by these findings, we next asked if the activation of JNK and ERK was downstream of H_2_O_2_ production. Indeed, we show that the presence of catalase (7000 units/ml) abrogated C1-induced JNK, ERK and c-Jun phosphorylation (Figure 6B). Similar results were obtained upon pre-incubation of cells with NAC or upon transient transfection of cells with human catalase gene (Figure 6B).

**Figure 7:**
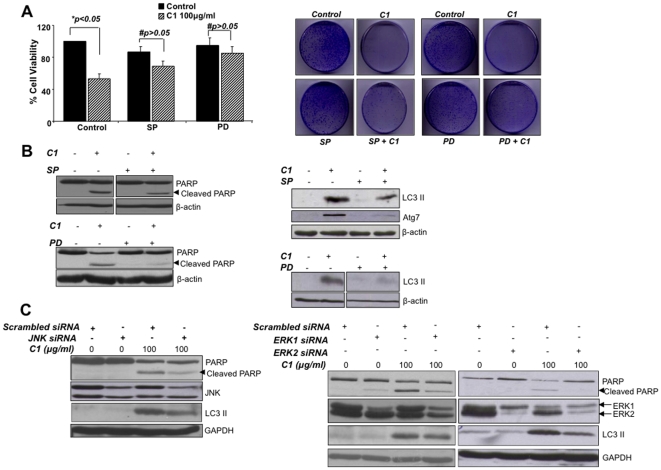
JNK and ERK activation are crucial mediators of autophagy and apoptosis induced by C1. Cells were pre-incubated with SP600125 (SP; 5 µM) or PD98059 (PD; 20 µM) for 1 hour before treatment with 100 µg/ml of C1 for 24 hours and (**Ai**) cell survival (MTT assay) and (**Aii**) tumor colony formation were determined. Total cell lysates from these cells were immunoblotted for (**Bi**) cleavage of PARP and (**Bii**) LC3 II formation and Atg7 expression. (**C**) Cells were transiently transfected with siRNA against JNK (**Ci**), ERK1/ERK2 (**Cii**) for 48 hours followed by exposure to C1 (100 µg/ml) for 24 hours. Lysates were then probed for PARP and LC3II.

To decipher the role of ERK and JNK activation in the two pathways induced by C1, we investigated the effect of pharmacological inhibition of JNK or ERK as well as their gene knock down using siRNA. Interestingly, pharmacological inhibition of JNK (SP600125) or ERK (PD98059) significantly rescued tumor cells from the short-term (over 24 hours) and long-term (colony forming ability) cytotoxic effects of C1 (Figure 7A), and clearly blocked apoptotic signaling as evidenced by the inhibition of PARP cleavage (Figure 7Bi). Moreover, pre-incubation of cells with either of the inhibitors significantly blocked LC3II formation induced by C1 (Figure 7Bii). Lastly, siRNA-mediated gene silencing of the specific MAPKs showed that knockdown of *ERK1*, *ERK2* and *JNK* inhibited PARP cleavage, while si*ERK2* and si*JNK* rendered tumor cells more resistant to autophagy as assessed by LC3II accumulation (Figure 7C). Taken together, these findings clearly highlight the involvement of ERK and JNK in the simultaneous triggering of drug-induced autophagy and apoptosis and strongly implicate ROS in this signaling pathway.

## Discussion

Here we report a novel mechanism of ROS-induced cell death with hallmarks of both autophagy and apoptosis in human tumor cell lines and in primary cells. Intriguingly, pre-treatment of cells with the pan-caspase inhibitor, zVAD-fmk, failed to rescue colony formation, despite blocking apoptotic features. Similarly, the necrosis inhibitor, necrostatin, had no effect on drug-induced cell death, thereby suggesting the existence of alternative cell death mechanism(s) in conjunction with apoptosis. Interestingly, electron microscopic and biochemical analyses revealed a morphological phenotype consistent with autophagy. Of note, neither the pharmacological inhibition of apoptosis (zVAD-fmk) nor autophagy (3-MA) was able to salvage cells from the effect of ROS inducing small molecule compound, C1, suggesting that both pathways were essential in death execution.

### Autophagic signaling induced by C1 follows a non-canonical pathway

The tumor suppressor protein Beclin1 has been shown to play a critical role in autophagy execution and its knockdown blocks autophagic cell death[39]. However, using a model of neurotoxin-induced cell death, Zhu *et al* showed that autophagic vacuolization was independent of Beclin1[40]. Similarly, in a breast cancer model, the phytoalexin resveratrol was found to induce non-canonical autophagy, which was dependent on inhibition of mTOR signaling[34]. Our data clearly indicate that gene silencing of *Beclin1* neither inhibited autophagy nor rescued cells from C1-induced cell death. Although, the functional relevance of autophagy as a cell survival response or a death execution mechanism is still being debated, recent evidence tends to favor the model whereby autophagy in the non-canonical settings is invariably associated with cell death[34]. In addition to the redundancy of Beclin1, the requirement of the class III PI3 kinase was also questionable as inhibition of its activity by 3-MA did not significantly rescue autophagic phenotype in C1-treated cells. This is also in line with the observations reported with resveratrol-induced autophagy[34]. Contrary to the effect of *Beclin1* knockdown, silencing of *Ulk1* (homolog of yeast Atg 1) or *ATG7* impeded autophagic signaling, thus providing evidence that these two proteins remained as essential mediators even in non-classical autophagy. However, it remains to be determined whether the multi-protein complex in the vesicle nucleation step is still being formed, regardless of the redundancy of Beclin1 in C1-induced autophagy. It should also be pointed out that as the autophagic signal did not involve the intermediacy of Beclin1, overexpression of the anti-apoptotic protein Bcl-2, which interacts with Beclin1 to block autophagy[41], had no effect on autophagy in our system (data not shown).

### Induction of early, complete autophagy and late stage, impaired autophagy in C1-treated cells

Autophagy is a dynamic, multi-step process, in which the disruption of autophagy could alter the steady-state balance of this process. In other words, accumulation of autophagosomes could either indicate increased autophagic flux or defective autophagy. Here we demonstrate an early increase in autophagic flux using lysosomal inhibitors as well as the assessment of p62/SQSTM1 accumulation. This phenomenon of complete/effective autophagy was initiated at an early stage upon C1 treatment and preceded lysosomal rupture. Late stage autophagy, however, occurs in a manner correlated with reduced autophagic clearance. This could be due, in part, to lysosomal permeabilization, which could impair autophagosome-lysosomal fusion thus blocking autophagy. Regardless, the early induction of autophagy in this system did contribute to the cell death execution as Atg7 silencing effectively abrogated cell death induced by C1. In this regard, our study adds to the increasing evidence of autophagy as an effective cell death trigger. Our findings support the notion that excessive increment of autophagosomes and p62 accumulation in the cells could represent a potent trigger for autophagic cell death to occur. On the other hand, reduced autophagic flux could be an effective trigger for irreversible cell death executed by apoptosis, which could override the death signals initiated by autophagy. Lysosomal rupture may be a critical switch of an incomplete autophagy to the induction of effective apoptosis by the release of pro-death lysosomal proteases of the cathepsin family. Another possible mediator for autophagic to apoptosis switch could be p62 accumulation, which has been shown to activate cell death[42].

### Autophagy and apoptosis are mutually exclusive but controlled by upstream ROS production

In several settings, autophagic signaling sets the stage for apoptosis to occur, while in others inhibition of autophagy triggers an apoptotic cell death program[43], [44]. Our data show that C1-induced cell death could be orchestrated through multiple signals that are independent of each other. Although apoptosis inhibitors effectively blocked caspase-dependent cell death, there was virtually no effect on the autophagic pathway. Reciprocally, gene silencing of *ATG7* or *ATG1* reduced the extent of autophagic induction, but apoptotic signaling remained unaffected. While the downstream signaling for each pathway appears to be autonomous, the upstream trigger controlling the induction of each of these signals is an early increase in intracellular ROS production, which drives ERK and JNK activation.

Classically, autophagy is described as a cellular clearance mechanism to remove damaged organelles and protein aggregates and thus serves as a cytoprotective mechanism to counteract oxidative stress in cells[22]. In yeast, mitophagy occurs as a response to nitrogen starvation and is mediated through a regulator of oxidative stress *Uth1* gene[45]. On the other hand, reports have also shown that ROS could serve as signaling molecules that directly or indirectly activate autophagy. To that end, it has been shown that induction of autophagy results in selective degradation of catalase, leading to accumulation of mitochondrial ROS and ultimately cell death[27]. In a separate study, TNF-α was shown to increase the expression of Beclin1 by a ROS-dependent mechanism[46]. Our data lend support to these observations by demonstrating the presence of mitochondrial-derived ROS in C1-induced autophagy, as well as the inhibitory effect of H_2_O_2_ scavenger, catalase. To provide a link between ROS production and autophagy, we highlighted its ability to activate two members of the MAP kinase family, JNK and ERK. Indeed, LC3II accumulation could be abolished through the exogenous addition of, or transient transfection with human catalase, suggesting a major signaling involvement of H_2_O_2_ in autophagy. This is consistent with a previous report on TNF-α induced accumulation of H_2_O_2_, which was shown to be responsible for autophagic cell death[46]. Indeed, H_2_O_2_ was also found to be an important mediator in starvation-induced autophagy, through its activity in regulating Atg4 protease[20]. Therefore, it appears that redox-regulation of autophagy is largely dependent on the magnitude and the rate of ROS accumulation.

### ROS-dependent ERK and JNK activation is responsible for autophagy and cell death

ROS has been shown as a classical inducer of the MAPK family members, through a variety of mechanisms including activation of MAP3K, such as ASK1 and oxidation of MKPs[6], [47]. Activation of the JNK and ERK signaling pathways by ROS represents a novel mechanism of autophagic induction. Inhibition of JNK and ERK activation by their pharmacological inhibitor, SP600125 and PD98059, respectively, resulted in the reduction of LC3II protein expression. Similarly, gene knock-down studies underscore the crucial involvement of JNK activation in the autophagic response triggered upon drug exposure. Interestingly pre-treatment of cells with cyclohexamide and actinomycin D reverted the increase in LC3II accumulation in C1-treated cells (data not shown), indicating that the increased accumulations of LC3II might involve protein synthesis. Of particular note, we show for the first time that JNK inhibition could effectively block the induction of Atg7 upon drug exposure of cells. We thus speculate that autophagy-inducing activity of JNK could be a function of its ability to induce the expression of Atg 7, a crucial mediator of autophagosome formation. To that end, JNK has been implicated in various models of autophagy, such as in response to serum starvation, cytokines, growth factor withdrawal and neurotoxic drugs[48], [49], [50], [51]. The myriad ways of JNK induction in autophagy may indicate that JNK is a core component in autophagic signaling pathway. In contrast, the mechanisms of ERK involvement in autophagy have not been extensively studied. In a neuroblastoma model, ERK 1/2 activation was reported to be critical in neurotoxin-induced autophagic cell death[40]. The activity of ERK and JNK were also found to be important in TNF-α induced apoptotic and autophagic cell death in L929 cells[19]. In this study, pharmacological inhibition of ERK or its gene knock-down almost completely abrogated the autophagic response triggered by C1, implicating ERK in the induction of autophagy. It remains to be seen whether the association of ERK activation with autophagy is a function of its ability to activate downstream transcription factors or an extra-nuclear function of ERK.

Similar to autophagy, the involvement of MAPKs in apoptosis have been demonstrated in a variety of model systems, including cytokines activation, oxidative stress, irradiation, and death receptor activation[35], [52], [53], [54]. Apart from transcriptional activation of c-Jun leading to induction of apoptotic genes such as Fas-L[55], transcriptional independent role of JNK in apoptosis has also been documented. This is largely via phosphorylation of pro or anti-apoptotic proteins such as p53 and Bcl-2[2]. We found that inhibition of ERK and JNK activation also attenuated apoptotic cell death. This was shown by inhibition of sub-G1 population, colony formation and cell death by ERK and JNK inhibitors as well as by siRNA directed against ERK1/2 and JNK. Thus, we provide evidence that ERK and JNK represent important mediators in controlling both signaling cascades of autophagy and apoptosis. Interestingly, it has been shown that JNK phosphorylation on Bcl-2 serves to promote autophagy and cell survival during early time points while delayed activation of JNK is a signal for apoptosis to occur[56]. In accordance with these findings, we demonstrate sustained and prolonged JNK activation, which may account for the differential cellular response brought about by C1. It is likely that early JNK activation signals for autophagy as an adaptive response to cellular stress while at the later time point when the cells are overwhelmed by cytotoxic stimuli, JNK activation serves to switch the cells towards apoptotic cell death. The observation that ERK and JNK control both autophagy and apoptosis could be of relevant to treatment of cancers where one of the death signaling machineries could be defective or deficient.

Taken together, these data underscore the tremendous potential of this small molecule compound for enhancing our understanding of the intricate complexities between different networks of cell death as well as for the therapeutic induction of cell death in tumors that are responsive to autophagic and/or apoptosis stimuli.

## Supporting Information

Figure S1 (A) MCF-7 cells were pre-incubated with necrostatin-1 and then treated with C1 (100 µg/ml) or TNFα (100 µg/ml). Viability was assessed by the MTT assay. (B) HCT116 cells were pre-treated with 3MA (5 mM dissolved in sterile distilled water at stock concentration of 400 mm and heated) for 2 hours followed by exposure to C1 (100 µg/ml) or C2-Ceramide (100 µM) for 24 hours. Lysates were probed for LC3II. (C) Cells were transiently transfected with siRNA against Beclin-1 and incubated in normal McCoy5A medium or amino acid and serum free medium (EBSS) for 6 hours. (D) Cells were pre-treated for 1 hour with DDC (100 µM) or SOD (5000 units/ml) and intracellular H2O2 was determined using the redox sensitive probe DCHF-DA as described in Materials and Methods. (E) Cells were pre-treated with DDC (100 µM) or SOD (1000 units/ml) for an hour prior to 24 hours exposure to C1 (100 µg/ml). Lysates obtained were probed for LC3II and cleavage of PARP. (F) Acridine orange staining was done as described in Materials and Methods following exposure of cells for 6 hours to 100 µg/ml of C1. (G) Cells were pre-treated with lysosomal inhibitors E64D and Pepstatin A (both 10 µg/ml) and then followed by exposure to C1100 µg/ml for 24 hours and 6 hours. Methanol was used as a vehicle control in this experiment. Cells were then lysed and blotted for LC3II. (H) Cells were exposed to C1 100 µg/ml for 2 to 24 hours and whole cell lysates were subjected to SDS-PAGE Western blotting for LC3II, PARP cleavage and SQSTM1.
(3.03 MB TIF)
