# Correction: Simultaneous Induction of Non-Canonical Autophagy and Apoptosis in Cancer Cells by ROS-Dependent ERK and JNK Activation

**DOI:** 10.1371/journal.pone.0159352

**Published:** 2016-07-08

**Authors:** Chew Hooi Wong, Kartini Bte Iskandar, Sanjiv Kumar Yadav, Jayshree L. Hirpara, Thomas Loh, Shazib Pervaiz

The authors would like to correct Fig 5, as errors were introduced in the preparation of this figure for publication. In the middle panel of Fig 5C, the loading control blot was mistakenly duplicated as well as mislabeled β-actin instead of GAPDH. The authors have provided a corrected version of Fig 5 here that includes both a corrected blot and a corrected label. The authors confirm that these changes do not alter their findings. The authors have provided the underlying images as Supporting Information.

**Fig 5:**
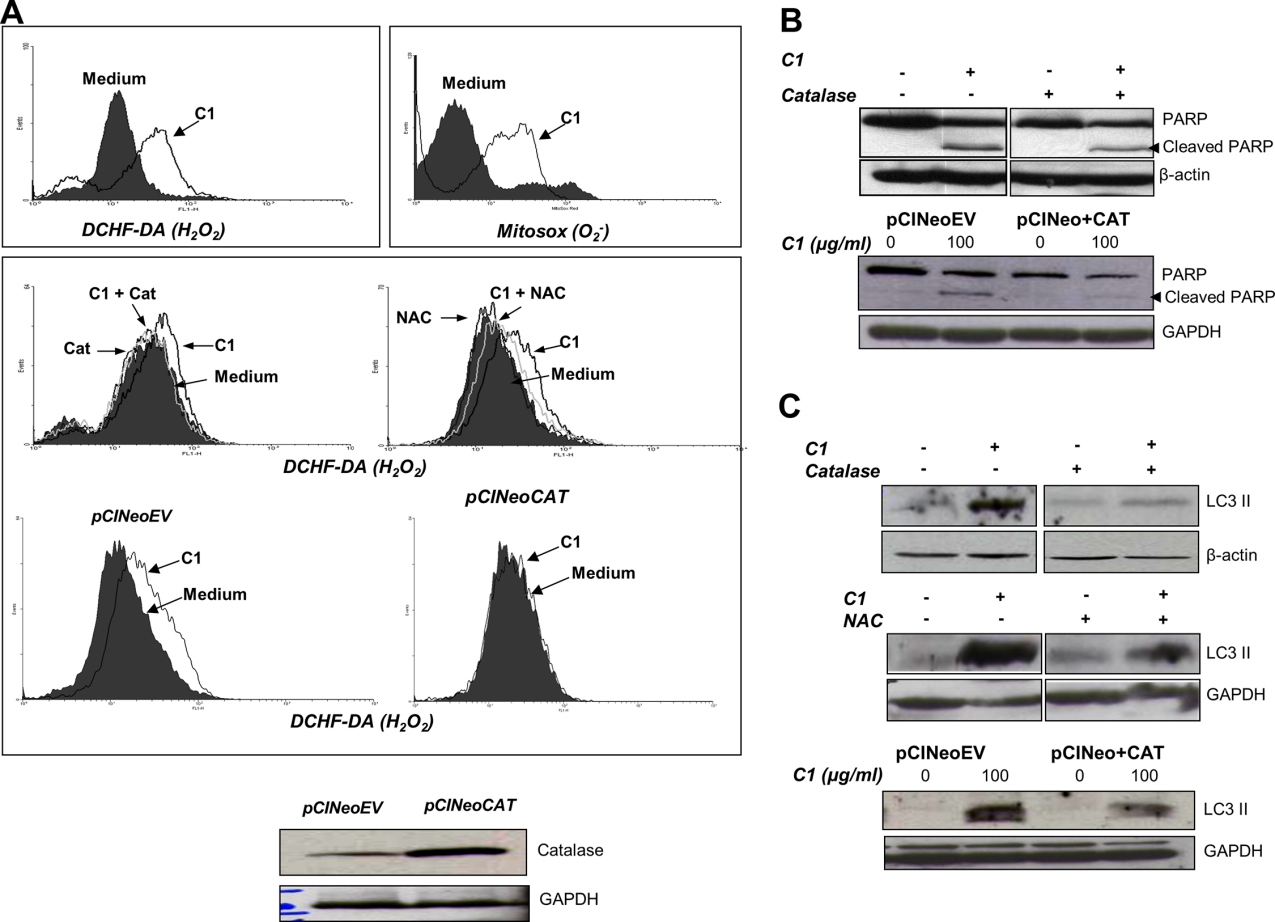
C1 induces early mitochondrial ROS production. In all, 1×10^6^ cells were incubated with 100 μg/ml of C1 for 3 hours and **(Ai)** intra-mitochondrial O_2_^-^ was determined using the fluorescent dye MitoSox™ RED Mitochondrial O_2_^-^ Indicator and intracellular H_2_O_2_ was detected by DCHF-DA loading and analyzed by flow cytometry. **(Ai)** Cells were pre-incubated with catalase (7000 units/ml) or NAC (200 μM) for 1 hour before treatment with C1 (100 μg/ml for 3 hours) and intracellular H_2_O_2_ was determined. **(Aii)** Cells were transiently transfected with 8 μg of pCINeoEV or pCINeo+CAT for 48 hours **(Aiii)** and treated with C1 (100 μg/ml for 3 hours) and intracellular H_2_O_2_ was determined **(Aii).** Cells were pre-incubated with catalase (7000 units/ml for 1 hour) or were transiently transfected with pCINeoEV or pCINeo+CAT before exposure to C1 (100 μg/ml for 24 hours), and total cell lysates were immunoblotted for **(B)** PARP cleavage and **(C)** LC3II accumulation.

## Supporting Information

S1 File Raw blots used to create Fig 5. (PPTX)
