# Supplementary figures and images for: Simultaneous Induction of Non-Canonical Autophagy and Apoptosis in Cancer Cells by ROS-Dependent ERK and JNK Activation
Source: PLoS One. 2010 Apr 2;5(4):e9996. doi: 10.1371/journal.pone.0009996 (PMC2848860; doi:10.1371/journal.pone.0009996)

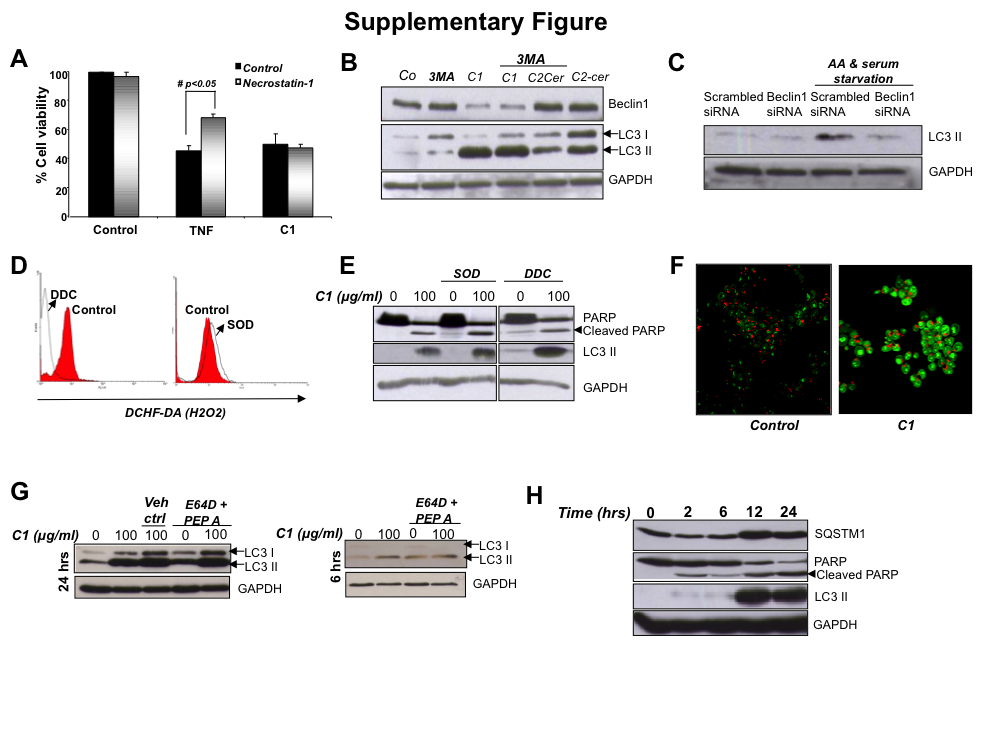

Supplement: Figure S1 — (A) MCF-7 cells were pre-incubated with necrostatin-1 and then treated with C1 (100 µg/ml) or TNFα (100 µg/ml). Viability was assessed by the MTT assay. (B) HCT116 cells were pre-treated with 3MA (5 mM dissolved in sterile distilled water at stock concentration of 400 mm and heated) for 2 hours followed by exposure to C1 (100 µg/ml) or C2-Ceramide (100 µM) for 24 hours. Lysates were probed for LC3II. (C) Cells were transiently transfected with siRNA against Beclin-1 and incubated in normal McCoy5A medium or amino acid and serum free medium (EBSS) for 6 hours. (D) Cells were pre-treated for 1 hour with DDC (100 µM) or SOD (5000 units/ml) and intracellular H2O2 was determined using the redox sensitive probe DCHF-DA as described in Materials and Methods. (E) Cells were pre-treated with DDC (100 µM) or SOD (1000 units/ml) for an hour prior to 24 hours exposure to C1 (100 µg/ml). Lysates obtained were probed for LC3II and cleavage of PARP. (F) Acridine orange staining was done as described in Materials and Methods following exposure of cells for 6 hours to 100 µg/ml of C1. (G) Cells were pre-treated with lysosomal inhibitors E64D and Pepstatin A (both 10 µg/ml) and then followed by exposure to C1100 µg/ml for 24 hours and 6 hours. Methanol was used as a vehicle control in this experiment. Cells were then lysed and blotted for LC3II. (H) Cells were exposed to C1 100 µg/ml for 2 to 24 hours and whole cell lysates were subjected to SDS-PAGE Western blotting for LC3II, PARP cleavage and SQSTM1. (3.03 MB TIF) [file pone.0009996.s001.tif]
