# Supplementary material for: Correction: Simultaneous Induction of Non-Canonical Autophagy and Apoptosis in Cancer Cells by ROS-Dependent ERK and JNK Activation
Source: PLoS One. 2016 Jul 8;11(7):e0159352. doi: 10.1371/journal.pone.0159352 (PMC4938624; doi:10.1371/journal.pone.0159352)
Supplement: S1 File — (PPTX) [file pone.0159352.s001.pptx]

## Slide 1
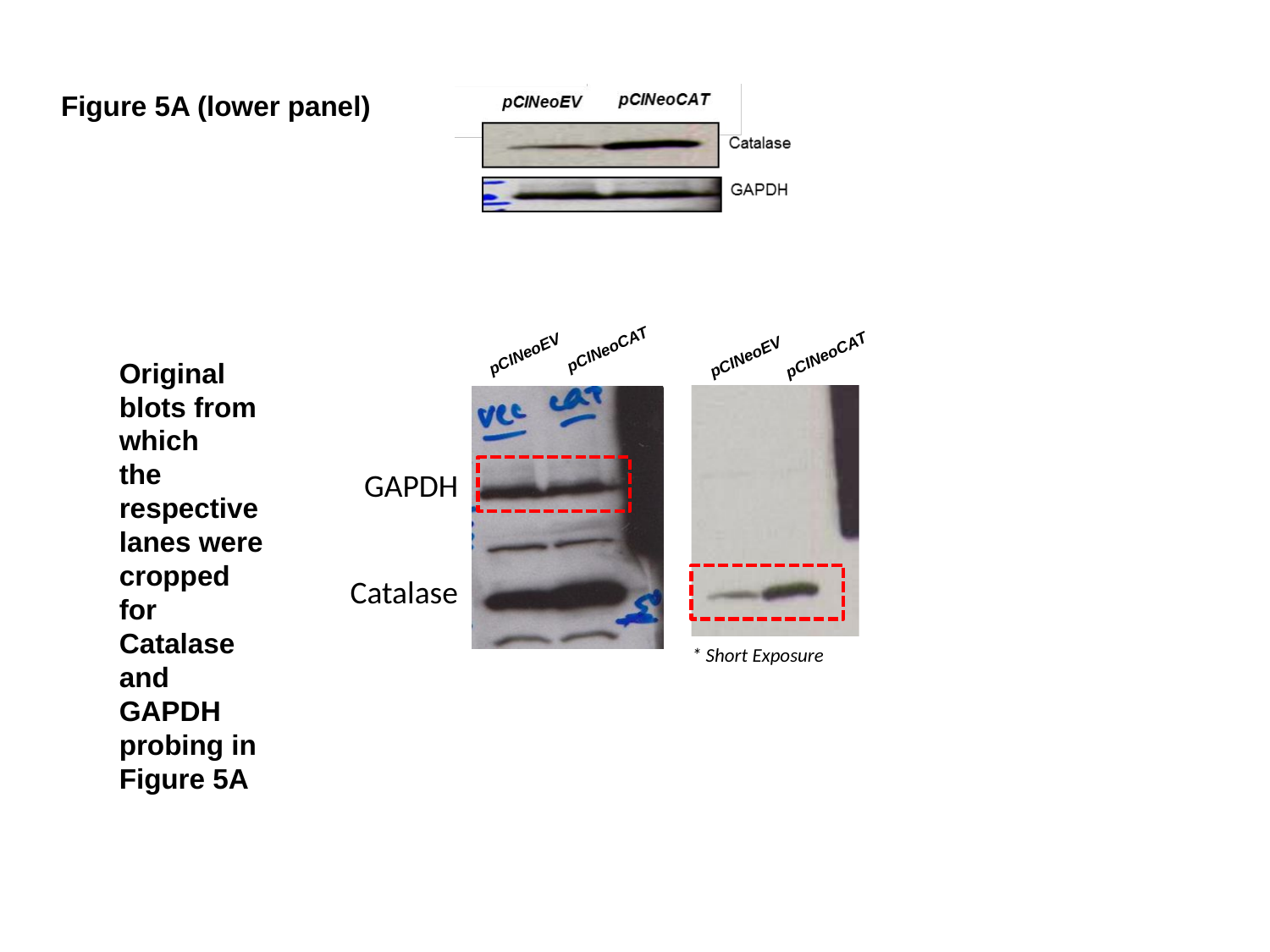

Figure 5A (lower panel)
pCINeoCAT
pCINeoEV
pCINeoCAT
pCINeoEV
Original blots from which
the respective lanes were cropped
for Catalase and GAPDH probing in Figure 5A
GAPDH
Catalase
* Short Exposure

## Slide 2
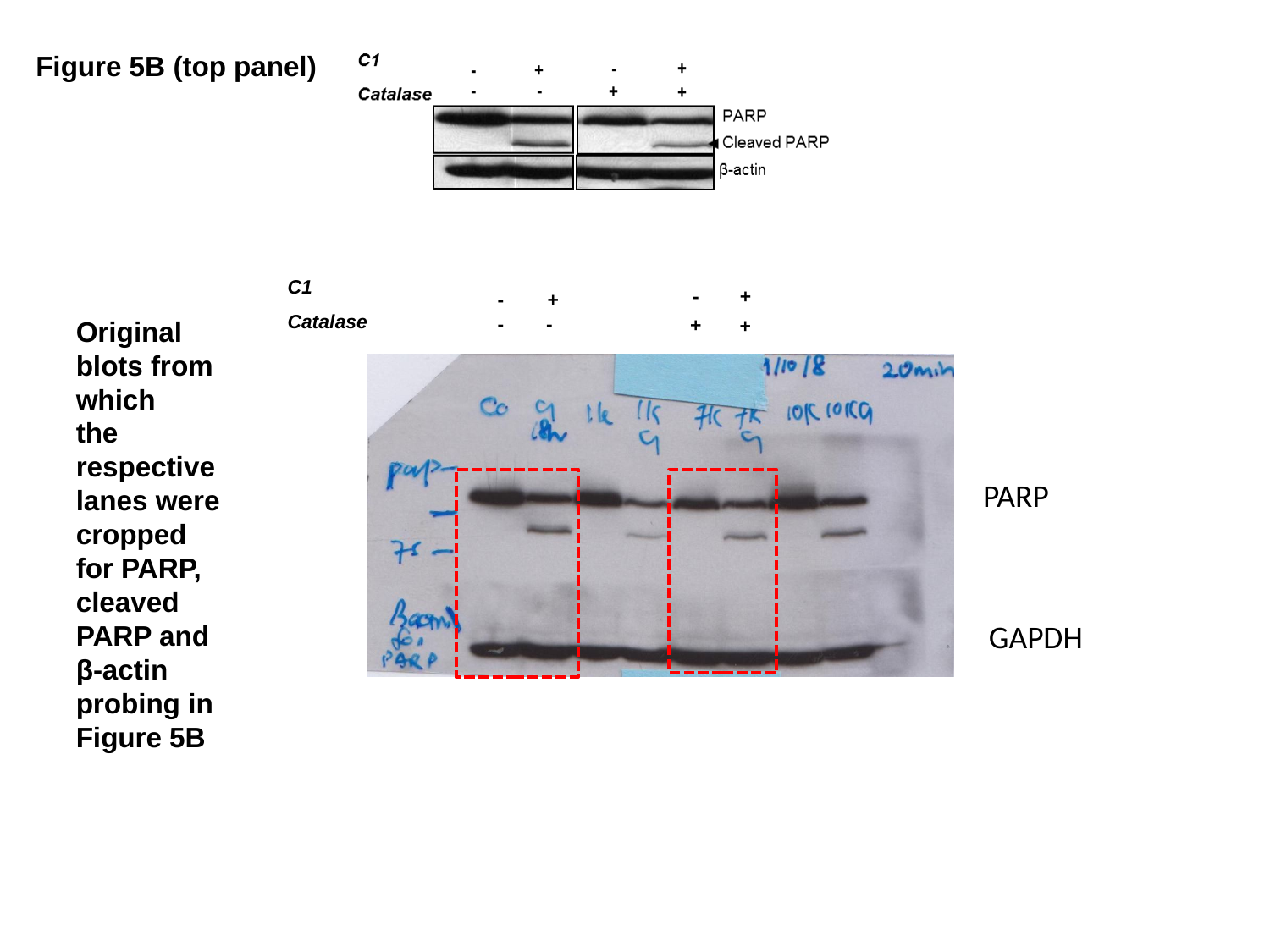

Figure 5B (top panel)
C1
Catalase
-
+
-
+
-
-
+
+
PARP
GAPDH
Original blots from which
the respective lanes were cropped
for PARP, cleaved PARP and β-actin probing in Figure 5B

## Slide 3
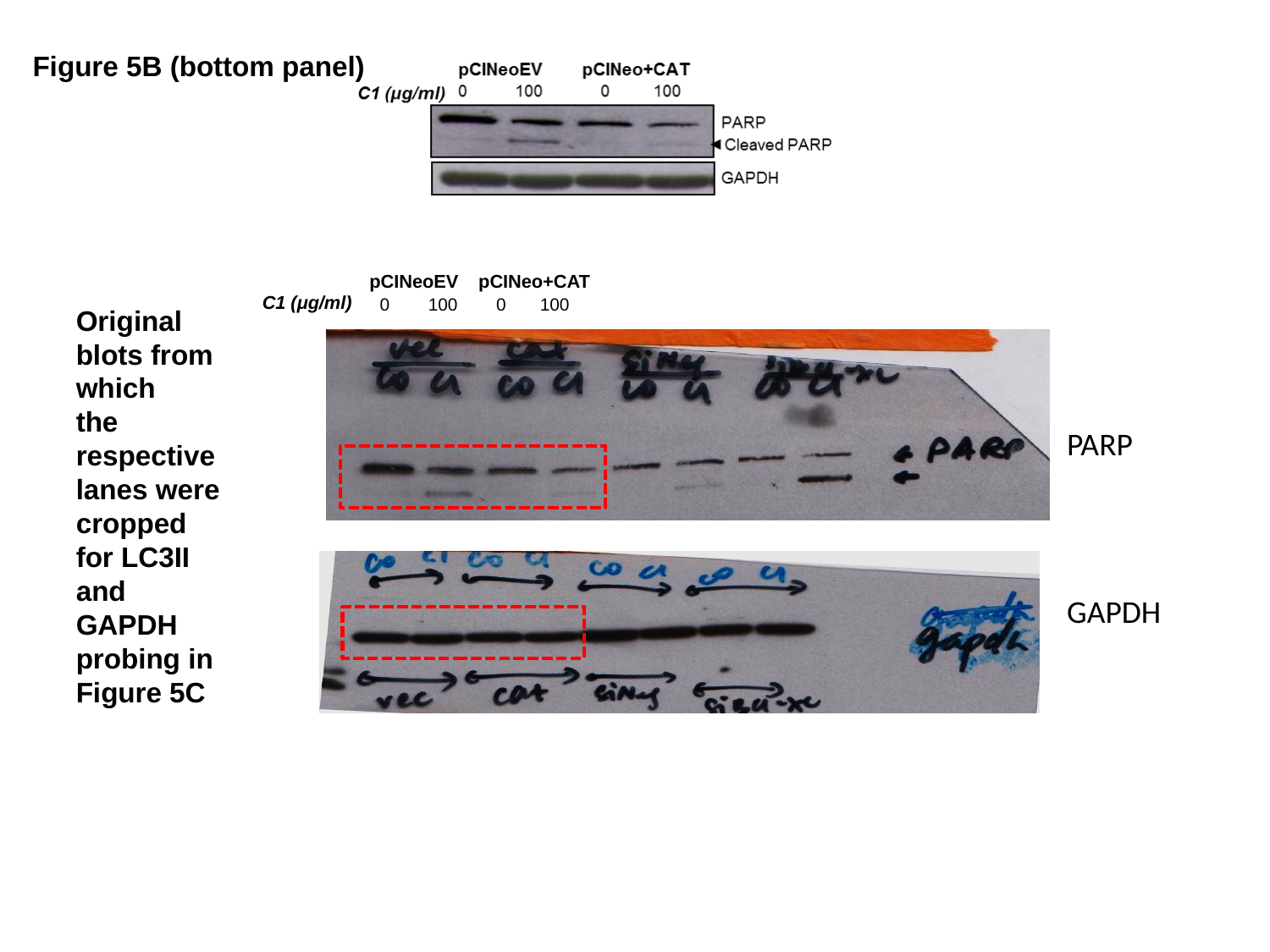

Figure 5B (bottom panel)
 pCINeoEV pCINeo+CAT
 0 100	 0 100
C1 (μg/ml)
Original blots from which
the respective lanes were cropped
for LC3II and GAPDH probing in Figure 5C
PARP
GAPDH

## Slide 4
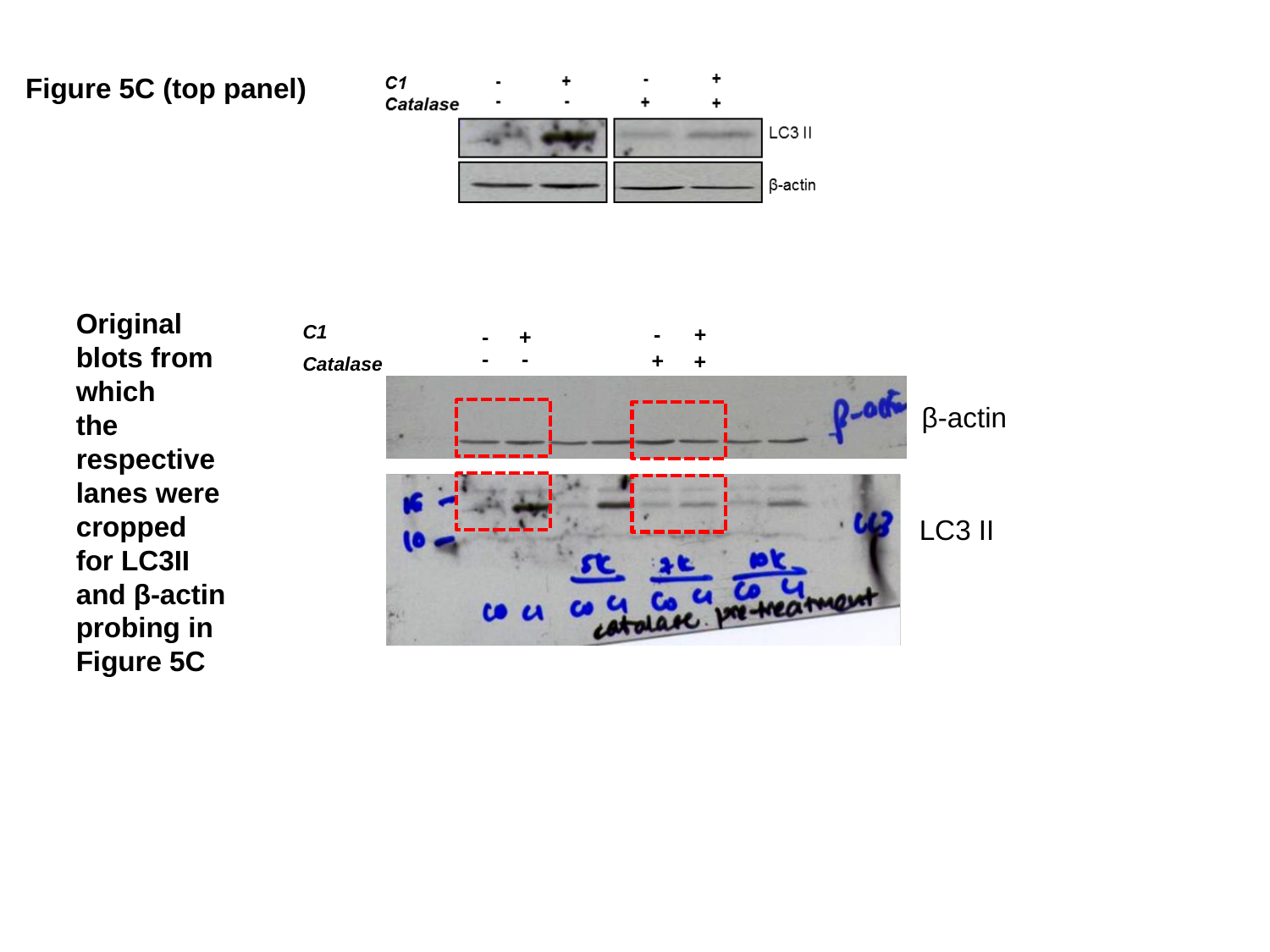

Figure 5C (top panel)
Original blots from which
the respective lanes were cropped
for LC3II and β-actin probing in Figure 5C
-
+
-
+
-
-
+
+
C1
Catalase
β-actin
LC3 II

## Slide 5
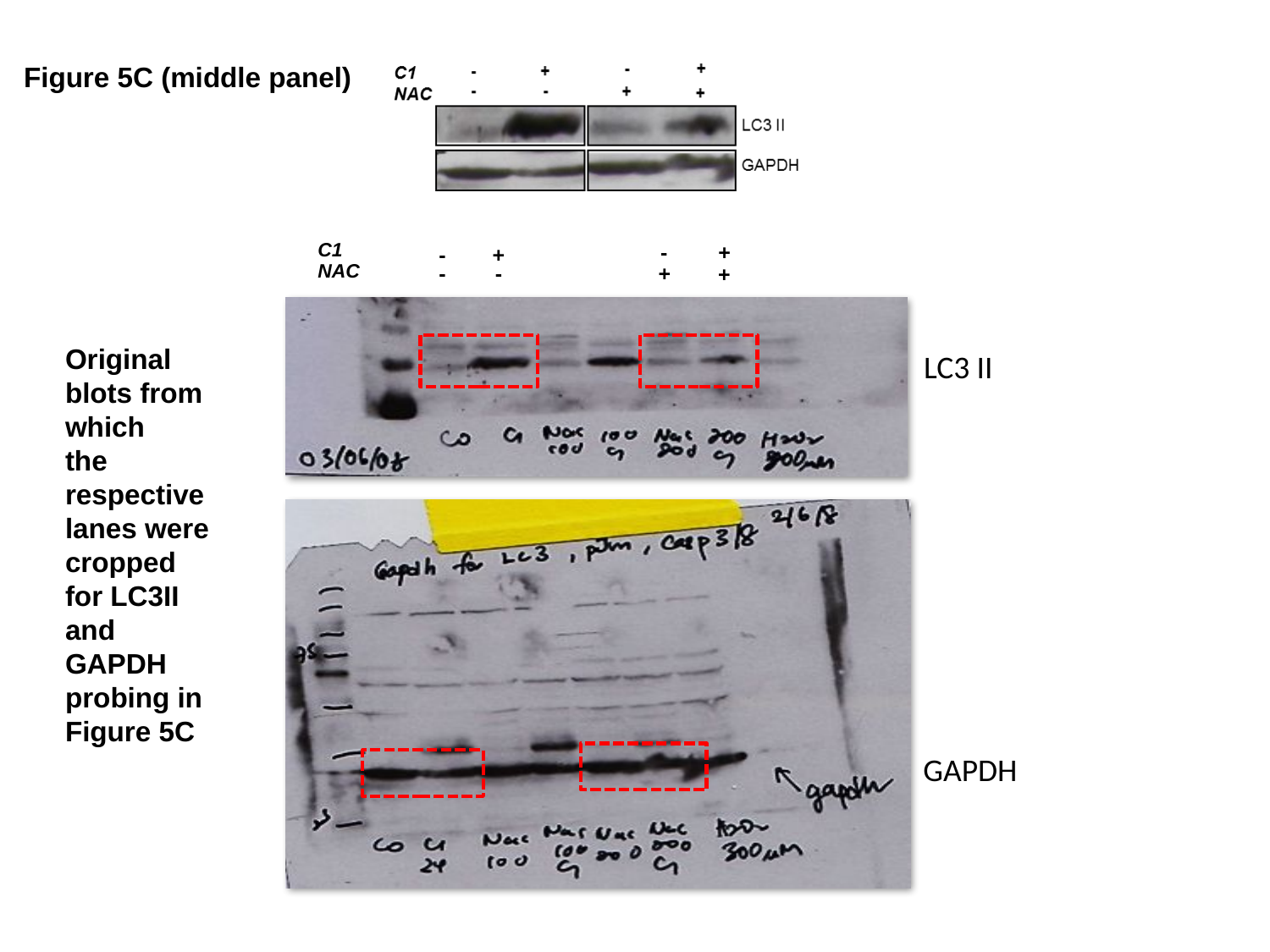

Figure 5C (middle panel)
-
+
-
+
-
-
+
+
C1
NAC
LC3 II
GAPDH
Original blots from which
the respective lanes were cropped
for LC3II and GAPDH probing in Figure 5C

## Slide 6
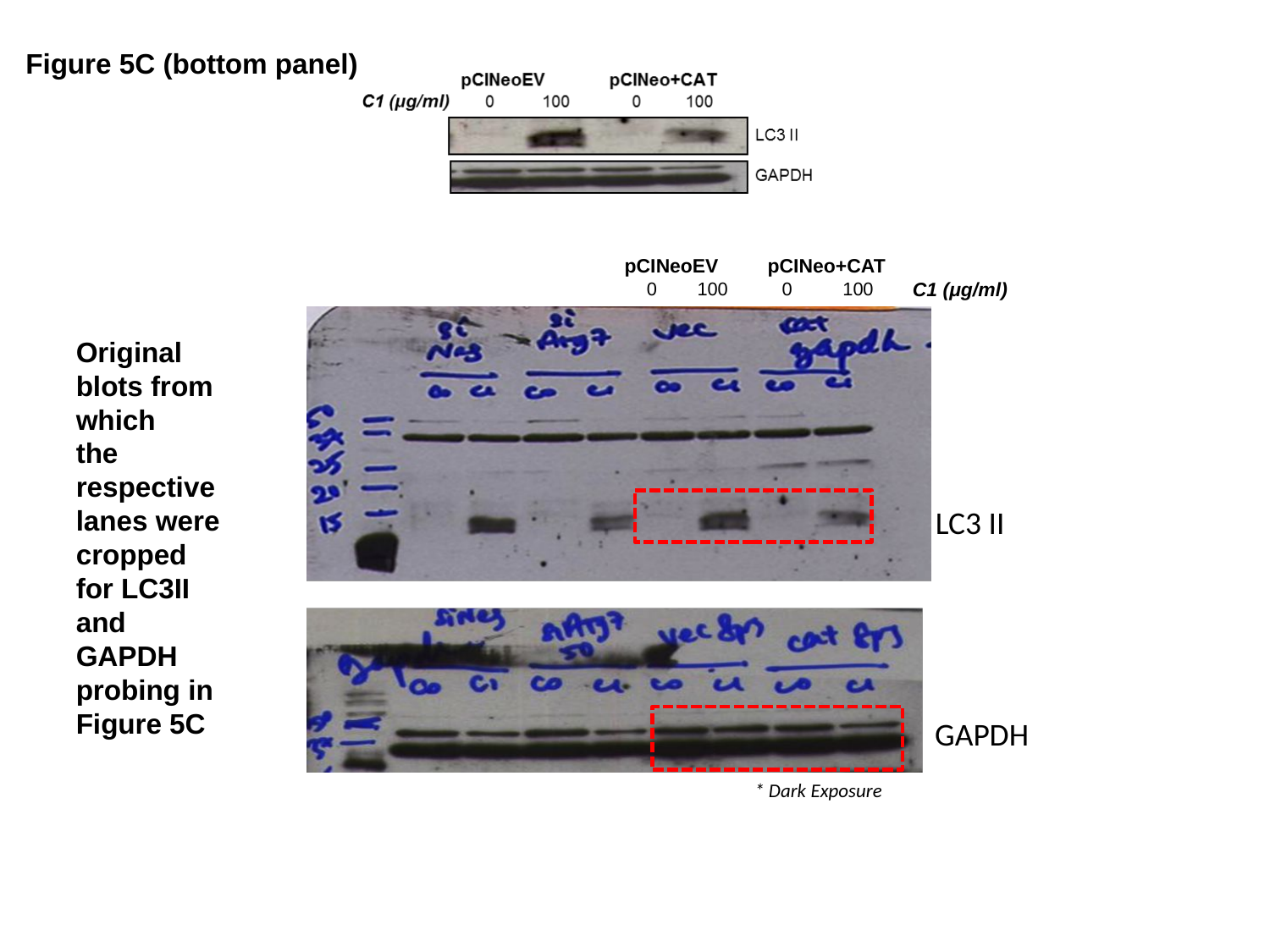

Figure 5C (bottom panel)
pCINeoEV pCINeo+CAT
 0 100	 0 100
C1 (μg/ml)
Original blots from which
the respective lanes were cropped
for LC3II and GAPDH probing in Figure 5C
LC3 II
GAPDH
* Dark Exposure
